# Comparing COVID-19 severity in patients hospitalized for community-associated Delta, BA.1 and BA.4/5 variant infection

**DOI:** 10.3389/fpubh.2024.1294261

**Published:** 2024-02-21

**Authors:** Maja Sočan, Maja Mrzel, Katarina Prosenc, Miša Korva, Tatjana Avšič-Županc, Mario Poljak, Maja M. Lunar, Tina Zupanič

**Affiliations:** ^1^National Institute of Public Health, Ljubljana, Slovenia; ^2^National Institute of Health, Environment and Food, Ljubljana, Slovenia; ^3^Institute of Microbiology and Immunology, Faculty of Medicine, University of Ljubljana, Ljubljana, Slovenia

**Keywords:** COVID-19, hospitalization, Delta, Omicron, BA.1, BA.4, BA.5

## Abstract

**Background:**

Despite decreasing COVID-19 disease severity during the Omicron waves, a proportion of patients still require hospitalization and intensive care.

**Objective:**

To compare demographic characteristics, comorbidities, vaccination status, and previous infections in patients hospitalized for community-associated COVID-19 (CAC) in predominantly Delta, Omicron BA.1 and BA.4/5 SARS-CoV-2 waves.

**Methods:**

Data were extracted from three national databases—the National COVID-19 Database, National Vaccination Registry and National Registry of Hospitalizations.

**Results:**

Among the hospitalized CAC patients analyzed in this study, 5,512 were infected with Delta, 1,120 with Omicron BA.1, and 1,143 with the Omicron BA.4/5 variant. The age and sex structure changed from Delta to BA.4/5, with the proportion of women (9.5% increase), children and adolescents (10.4% increase), and octa- and nonagenarians increasing significantly (24.5% increase). Significantly more patients had comorbidities (measured by the Charlson Comorbidity Index), 30.3% in Delta and 43% in BA.4/5 period. The need for non-invasive ventilatory support (NiVS), ICU admission, mechanical ventilation (MV), and in-hospital mortality (IHM) decreased from Delta to Omicron BA.4/5 period for 12.6, 13.5, 11.5, and 6.3%, respectively. Multivariate analysis revealed significantly lower odds for ICU admission (OR 0.68, CI 0.54–0.84, *p* < 0.001) and IHM (OR 0.74, CI 0.58–0.93, *p* = 0.011) during the Delta period in patients who had been fully vaccinated or boosted with a COVID-19 vaccine within the previous 6 months. In the BA.1 variant period, patients who had less than 6 months elapsed between the last vaccine dose and SARS-CoV-2 positivity had lower odds for MV (OR 0.38, CI 0.18-0.72, *p* = 0.005) and IHM (OR 0.56, CI 0.37- 0.83, *p* = 0.005), but not for NIVS or ICU admission.

**Conclusion:**

The likelihood of developing severe CAC in hospitalized patients was higher in those with the Delta and Omicron BA.1 variant compared to BA.4/5.

## 1 Introduction

The COVID-19 pandemic has caused significant morbidity, mortality, and social disruption. COVID-19 severity is influenced by a combination of factors: demographic (age, sex), health status (comorbidities), immunity (vaccination and previous infections), availability of health care services (testing and early therapy), and virulence of the SARS-CoV-2 variants (1, 2).

The Omicron variant was first characterized in South Africa in mid-November 2021, where the lower COVID-19 disease severity and higher transmissibility of this variant were well documented (1, 3–6). Individuals with Omicron vs. non-Omicron infections had 80% lower odds of being admitted to hospital and when compared with Delta variant infections, Omicron infections were associated with a 70% lower odds of severe disease (3). Subsequent studies from different countries confirmed the South African experience with the first Omicron variants (BA.1, BA.1.1, and BA.2) (7–10), in which the proportion of confirmed COVID-19 cases admitted to hospitals and the in-hospital mortality were reduced (8). The Omicron variant was also associated with lower disease severity in children under five and older adults (11, 12). The risk of hospitalization was lower, but not uniformly so among all age groups, and a UK study found that the risk of hospitalization due to Omicron infection was not significantly different from that of Delta infection in individuals aged 0–9 years (13).

The lower severity of Omicron might be due to a large number of novel mutations that attenuated the virulence of this variant (14). Research showed that replication of the Omicron and Delta isolates was similar in human nasal epithelial cell cultures, but Omicron showed slower replication in lung and gut cells (15). In addition, the spike protein was cleaved less efficiently by Omicron compared to Delta. On the other hand, studies also showed that the BA.1 and BA.2 variants have intrinsically higher replication competence in the human upper respiratory tract (nasal and bronchi tissues) compared to previous variants. Omicron BA.2 has the ability to replicate at 33°C, which may contribute to increased transmission in the human population (16, 17). Moreover, with each wave of the pandemic natural or vaccine-acquired population immunity increased, resulting in greater protection against a more severe disease (18, 19).

Despite the reduced disease severity during subsequent Omicron waves, a proportion of patients still required hospitalization and intensive care. In this study we compared the demographic characteristics, comorbidities, vaccination status, and previous infections in patients hospitalized during the Delta, Omicron BA.1, and BA.4/5 SARS-CoV-2 waves. The aim of the study was to assess whether the characteristics of patients admitted for (rather than with) COVID-19 differed during the different waves.

## 2 Methods

### 2.1 Data sources

To study the differences in demographics, comorbidities, and COVID-19 vaccination history among hospitalized patients in the Delta, BA.1, and BA.4/5 waves in Slovenia, data were obtained from three national health electronic databases—the National COVID-19 Database, National Vaccination Register and National Registry of Hospitalizations, from the onset of the SARS-CoV-2 epidemic in Slovenia (March 4, 2020) to November 30, 2022.

The National COVID-19 Database is part of the National Notifiable Communicable Diseases Database and is linked to the Central Registry of Patient Data. The data-base records all laboratory-confirmed SARS-CoV-2 cases (symptomatic and asymptomatic) in Slovenia. According to the national definition, a confirmed case of SARS-CoV-2 infection is defined by a positive RT-PCR test or validated rapid antigen test (RAT). The following data were obtained from the National COVID-19 Database: age (in years), sex, and date of confirmed infections (primary infection and reinfections).

Data on COVID-19 vaccinations were extracted from the National Vaccination Register (eRCO, in Slovenian: Elektronski register cepljenih oseb, in English: Electronic Register of Vaccinated Persons). The data extracted from eRCO were the vaccination dates and the vaccine used.

**Figure 1:**
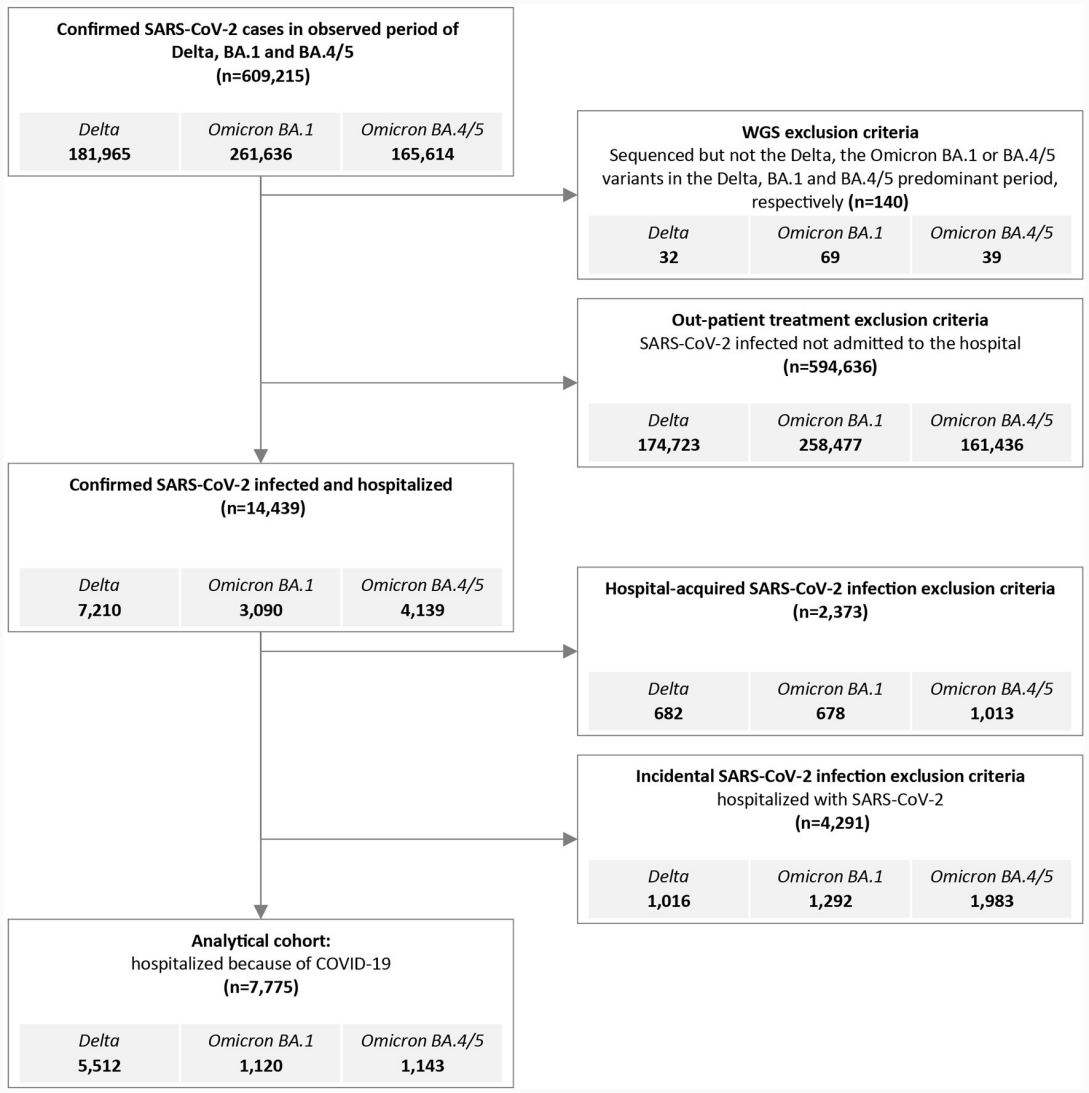
Flowchart outlining inclusion and exclusion criteria to generate the Delta, the Omicron BA.1 and BA.4/5 hospitalized cohort for analysis.

The National Registry of Hospitalizations (eSBO, in Slovenian: Elektronski sistem bolnišničnih obravnav, in English: Electronic Registry of Hospitalizations) was used to obtain data on hospitalization of laboratory confirmed SARS-CoV-2 cases. The following data were collected from the eSBO: main discharge and additional diagnoses, length of stay (LoS, in days), non-invasive ventilatory support (NiVS), intensive care unit treatment (ICU), mechanical ventilation (MV), and outcome (in-hospital mortality (IHM) or hospital discharge). Patients with hospital-acquired SARS-CoV-2 infection were excluded from the study. Patients admitted with asymptomatic or mild SARS-CoV-2 infection incidentally detected during SARS-CoV-2 screening at hospital admission were also excluded from the study. Only patients admitted to the hospital because of COVID-19 were included in the analysis (Figure 1). Database of Deceased Persons (in Slovenian: Zbirka podatkov o umrlih osebah) was used to verify intrahospital mortality.

Individual data in the national registries were linked by a unique personal identification number. The National COVID-19 Database, eRCO, eSBO, and Database of De-ceased Persons are managed by the National Institute of Public Health (NIPH) of Slovenia. Non-Slovenian residents who tested positive for SARS-CoV-2 in Slovenia but did not have a national personal identification number (transient visitors, irregular immigrants, etc.) were not included in the analysis.

To determine the prevalence of SARS-CoV-2 variants observed in the Slovenian population, we accessed the GISAID global database and extracted the corresponding prevalence of each variant. On this basis we identified periods when Delta, Omicron BA.1 and BA4/5 were the predominant variants circulating in Slovenia, determined by ≥95% of sequenced strains belonging to the same strain.

Between week 29/2021 and week 49/2021, the Delta variant accounted for 98.3% to 100% of the strains identified nationwide, and so hospitalized patients with confirmed SARS-CoV-2 infection during this period were considered to be infected with the Delta variant. Between week 3/2022 and week 5/2022, 95.0% to 96.2% of infections were due to the Omicron BA.1 variant, and patients diagnosed during this period were thus considered to be infected with the Omicron BA.1 variant. Omicron BA.4 and BA.5 co-circulated and were predominant between week 28/2022 and week 41/2022 (ac-counting for 95.7% to 99.3% of sequenced strains).

### 2.2 Definitions

For the classification of study participants, we used the following definitions:

#### 2.2.1 SARS-CoV-2 primary infection and reinfection

Primary infection—first notified infection with SARS-CoV-2 in the National COVID-19 Database confirmed by a positive RT-PCR or RAT.

Reinfection—SARS-CoV-2 infection that followed primary infection with an interval of ≥45 days.

#### 2.2.2 COVID-19 vaccination history

Hospitalized patients were divided in four groups:

(i) Non-vaccinated: Participants who had not received an anti-SARS-CoV-2 vaccine dose before hospitalization or had received only one dose of a two-dose schedule vaccine [the mRNA vaccines Comirnaty (Pfizer/BioNTech) or Spikevax (Moderna), the vector vaccine Vaxzevria (Astra-Zeneca)] or one dose of Jcovden/Janssen (Johnson & Johnson) within 14 days before hospitalization.

(ii) Partially vaccinated: Participants who had received only one dose of a vaccine with a two-dose schedule [mRNA vaccines Comirnaty (Pfizer/BioNTech) or Spikevax (Moderna), the vector vaccine Vaxzevria (Astra-Zeneca)] at least 14 days before hospitalization.

(iii) Fully vaccinated: Participants who had received one dose of Jcovden/Janssen vaccine (Johnson & Johnson) or both doses of a two-dose schedule vaccine [the mRNA vaccines Comirnaty (Pfizer/BioNTech) or Spikevax (Moderna), the vector vaccine Vaxzevria (Astra-Zeneca)] at least 14 days before hospitalization.

(iv) Vaccinated with additional dose: fully vaccinated participants who received at least one additional dose of an mRNA-based vaccine [Comirnaty (Pfizer/BioNTech) or Spikevax (Moderna)] at least 14 days before hospitalization.

**Figure 2:**
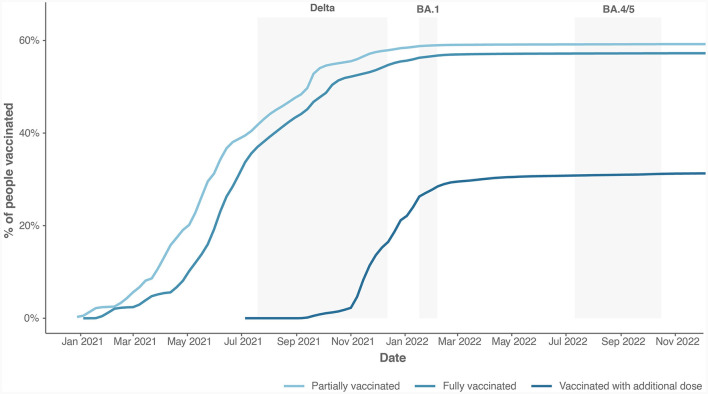
The cumulative vaccination coverage with all types of COVID-19 vaccines available in Slovenia.

We calculated the time (in days) that elapsed since the last dose of COVID-19 vaccine, considering only patients who were fully vaccinated (at least two doses for two-dose vaccine or one dose for single-dose vaccine). Figure 2 shows the cumulative vaccination coverage with COVID-19 vaccines in Slovenia. Overall, 59% of Slovenians received at least one dose of COVID-19 vaccine. Among them, over 97% are fully vaccinated. Within those vaccinated, 55% received booster shots. From the figure, we can observe that the coverage arrives at a plateau after March 2022, showing little gain in further coverage.

#### 2.2.3 Hospital admissions

Since the beginning of the COVID-19 epidemic in Slovenia (March 2020), patients admitted with acute respiratory symptoms were routinely tested for SARS-CoV-2 infection before hospitalization (if not already confirmed as a COVID-19 case before admission). In addition, screening for SARS-CoV-2 infection was performed in all asymptomatic individuals admitted to any hospital in the country (acute and planned admissions, admissions to maternity units and healthy parents/guardians admitted with their children etc.). Post-admission screening was performed in hospitalized patients who were in contact with a SARS-CoV-2 positive patient or health care worker or who developed symptoms consistent with COVID-19 during hospitalization.

Hospitalized SARS-CoV-2 positive patients were classified in three groups:

(i) Community-associated COVID-19 (CAC): Patients admitted for community-associated COVID-19 severe enough to warrant hospital admission, defined by COVID-19 being the main discharge diagnosis (ICD-10 classification B34.2) or if the main discharge diagnosis was acute respiratory infection caused by SARS-CoV-2 (the combination of any ICD-10 code for acute respiratory infection as the main discharge diagnosis and ICD-10 U07.1, B34.2, or B97.2 as an additional diagnosis).

(ii) Incidentally infected with SARS-CoV-2: Patients admitted primarily for an-other medical reason who were diagnosed with SARS-CoV-2 infection on admission or during hospitalization, but not severe enough to warrant admission – the main dis-charge diagnosis was any ICD-10 diagnosis except COVID-19.

(iii) Hospital-associated SARS-CoV-2: Hospital-associated SARS-CoV-2 infection defined by a difference of ≥5 days between the date of admission and the date of the positive SARS-CoV-2 RT-PCR or RAT after admission.

Only patients hospitalized for community-associated COVID-19 during the Delta, BA.1 and BA.5 periods were included in the study. Being a long-term care resident was not an exclusion criterion for a community-associated COVID-19 case.

We calculated the Charlson Comorbidity Index (CCI) using the original weights and the revised Quan weights (20). Patients were divided into four groups according to their CCI scores (0 CCI, 1 CCI, 2 CCI, and ≥3 CCI).

### 2.3 Statistical analysis

Descriptive analysis was performed to summarize the characteristics of hospitalized patients, with the results presented as the frequency and percentage for categorical variables and as the median and interquartile range (IQR) for continuous variables. Differences between variants were evaluated with the Chi-square test or Fisher's exact test for categorical variables, and with the Kruskal-Wallis test for non-normally distributed continuous variables.

Because of the large number of independent variables, multiple univariate logistic regressions were performed separately for each SARS-CoV-2 variant and each outcome to identify the most significant variables. These were then included in multivariate logistic regressions in which we analyzed patient outcomes (NiVS, ICU, MV, and IHM) by variant. Finally, to compare the severity of variants we performed multiple logistic regression for each outcome separately, while controlling for sex, age, vaccination, reinfection, and comorbidity index. The results of logistic regressions are presented as odds ratios, 95% confidence intervals, and associated *p*-values.

A two-sided *p*-value of <0.05 was considered statistically significant. Statistical analyses were performed using SPSS software, version 27 (IBM, Armonk, New York, United States) and R, version 4.1.3 (R Foundation for Statistical Computing, Vienna, Austria).

### 2.4 Ethics statement

The study was conducted in accordance with the Declaration of Helsinki and approved by De-ontological and Ethical Board, National Institute of Public Health, No. 631-21/2023-6 (013).

## 3 Results

A total of 609,215 people tested positive (RT-PCR: 396,591, RAT: 212,624) for SARS-CoV-2 in Slovenia during the Delta, BA.1 and BA.4/5 periods, and 34,674 (5.7%) samples were sequenced and reported to GISAID (Figure 1). We excluded 140 (0.02%) people with known SARS-CoV-2 sequence which differed from the predominant period variant. During the defined Delta, BA.1 and BA.4/5 periods a total of 7,210 (3.96%), 3,090 (1.18%), and 4,139 (2.50%) patients, respectively, were hospitalized with confirmed SARS-CoV-2 infection. The proportion of those who were treated in the hospitals for community associated COVID-19 (CAC), with community associated COVID-19 (or confirmed SARS-CoV-2 infection), or with SARS-CoV-2 hospital-associated infection (HAI) differed between variant periods (Figure 1). Furthermore, 15.2, 16.5, and 18.5% of SARS-CoV-2 HAI patients were admitted to psychiatric hospitals or rehabilitation units, respectively. The average length of stay in the abovementioned wards was longer and provided a greater chance of acquiring SARS-CoV-2 HAI.

Only patients hospitalized for CAC were included in the analysis. A total of 7,775 patients were included in the study: 5,512 (3.03%) infected with Delta, 1,120 (0.43%) with Omicron BA.1, and 1,143 (0.69%) with Omicron BA.4/5 variant. Fifteen patients were hospitalized twice during the study period, 11 patients with Delta, three patients with Omicron BA.1, and one with Omicron BA.4/5 primary infection. Reinfection of all 15 patients occurred with the Omicron BA.4/5 variants.

The characteristics of patients hospitalized for CAC by SARS-CoV-2 variant are shown in Table 1. There were more female than male patients hospitalized in the BA.4/5 variant period, while more male patients were hospitalized during Delta and Omicron BA.1 wave. The age distribution of patients admitted to hospitals was significantly different between the Delta wave and the BA.4/5 wave. In the BA.4/5 period, children and adolescents accounted for one in eight patients hospitalized for COVID-19, and patients ≥ 80 years accounted for nearly half of the COVID-19 admissions, whereas in the Delta wave most hospitalizations were among those aged 18-64 years. Long-term care residents presented 2.6, 11.3, and 8.9% of admissions in the Delta, BA.1, and BA.4/5 periods, respectively.

**Table 1:** Characteristics of analytical cohort, overall and by the Delta and the Omicron variants.

	**Overall, *N =* 7,775^a^**	**Delta, W29–W49 2021 *N =* 5,512^a^**	**Omicron BA.1, W3–W5 2022 *N =* 1,120^a^**	**Omicron BA.4/5, W28–W41 2022 *N =* 1,143^a^**	***p*-value^b^**
**Gender**					< 0.001
Female	3,623 (46.6%)	2,483 (45.0%)	517 (46.2%)	623 (54.5%)	
Male	4,152 (53.4%)	3,029 (55.0%)	603 (53.8%)	520 (45.5%)	
**Median age and interquartile range (in years)**	69 (55, 80)	66 (53, 78)	73 (60, 84)	78 (66, 85.5)	< 0.001
**Age**					< 0.001
0–17 years	347 (4.5%)	118 (2.1%)	86 (7.7%)	143 (12.5%)	
18–64 years	2,830 (36.4%)	2,438 (44.2%)	268 (23.9%)	124 (10.8%)	
65–79 years	2,471 (31.8%)	1,752 (31.8%)	372 (33.2%)	347 (30.4%)	
≥80 years	2,127 (27.3%)	1,204 (21.9%)	394 (35.2%)	529 (46.3%)	
**Care home resident**					< 0.001
Others	7,402 (95.2%)	5,367 (97.4%)	994 (88.8%)	1,041 (91.1%)	
Long term care residents	373 (4.8%)	145 (2.6%)	126 (11.2%)	102 (8.9%)	
**Vaccination**					< 0.001
Non-vaccinated	5,022 (64.6%)	3,915 (71.0%)	670 (59.8%)	437 (38.2%)	
Partially vaccinated	114 (1.5%)	81 (1.5%)	27 (2.4%)	6 (0.5%)	
Fully vaccinated	1,973 (25.4%)	1,500 (27.2%)	277 (24.7%)	196 (17.2%)	
Vaccinated with additional dose(s)	666 (8.5%)	16 (0.3%)	146 (13.1%)	504 (44.1%)	
**Time since last vaccination (days) if vacc. completely or w. add. dose/-s**					< 0.001
Non-vaccinated or partially vaccinated	5,136 (66.1%)	3,996 (72.5%)	697 (62.2%)	443 (38.8%)	
14–180 days since last vaccination	1,031 (13.3%)	740 (13.4%)	267 (23.8%)	24 (2.1%)	
181–270 days since last vaccination	966 (12.4%)	676 (12.3%)	89 (8.0%)	201 (17.6%)	
271+ days since last vaccination	642 (8.2%)	100 (1.8%)	67 (6.0%)	475 (41.5%)	
**Reinfection**					< 0.001
Primary infection	7,563 (97.3%)	5,488 (99.6%)	1,077 (96.2%)	998 (87.3%)	
Reinfection	212 (2.7%)	24 (0.4%)	43 (3.8%)	145 (12.7%)	
**Time since previous positive result (in days)**	352 (251, 574.5)	301 (275.8, 329)	403 (382, 439)	324 (221, 610)	0.012
**Charlson comorbidity index with original weights**					< 0.001
0 CCI	5,200 (66.9%)	3,841 (69.7%)	707 (63.1%)	652 (57.0%)	
1 CCI	1,446 (18.6%)	974 (17.7%)	212 (18.9%)	260 (22.8%)	
2 CCI	661 (8.5%)	412 (7.5%)	124 (11.1%)	125 (10.9%)	
3+ CCI	468 (6.0%)	285 (5.1%)	77 (6.9%)	106 (9.3%)	
**Charlson comorbidity index with revised Quan weights**					< 0.001
0 CCI	5,883 (75.7%)	4,363 (79.2%)	784 (70.0%)	736 (64.4%)	
1 CCI	625 (8.0%)	416 (7.5%)	91 (8.1%)	118 (10.3%)	
2 CCI	882 (11.3%)	517 (9.4%)	175 (15.6%)	190 (16.6%)	
3+ CCI	385 (5.0%)	216 (3.9%)	70 (6.3%)	99 (8.7%)	

a n (%); Median (interquartile range).

b Pearson's Chi-squared test; Kruskal-Wallis rank sum test; Fisher's exact test.

As expected, the proportions in vaccination status changed—a decrease in the proportion of unvaccinated/partially vaccinated people was observed (72.5, 62.2, and 38.8%, in the Delta, BA.1, and BA.4/5 period, respectively), and a high proportion (44.1%) of hospitalized patients for COVID-19 in the BA.4/5 period had already received an additional dose of the vaccine. Most patients who were fully or additionally vaccinated before hospital admission in the Delta or BA.1 period (93.4 and 84.2%, respectively) received their last dose of vaccine 14 to 270 days before the positive RT-PCR result. As expected, the number of COVID-19 patients in the BA.4/5 period who received their last vaccine dose in the 14- to 270-day period was much lower (32.1%), with only 3.4% of patients vaccinated in the last 6 months before hospitalization.

The proportion of re-infected hospitalized CAC patients increased from the Delta period to BA.4/5 period. The longest median time from initial infection to reinfection which resulted in hospitalization for CAC was in BA.1 period (403 days, interquartile range 382–439) (Table 1).

A statistically significant difference in Charlson Comorbidity Index (CCI) distribution was observed between the Delta and BA.4/5 period. The number of COVID-19 patients with a CCI score of zero decreased and the number of patients with a CCI score ≥3 increased. Using Quan modification of CCI, similar results were obtained (Table 1) with lower proportion in CCI value of 1. The list of co-morbidity diagnoses, frequencies, and *p*-values for patients in the Delta, BA.1, and BA.4/5 periods are presented in Supplementary Table 1.

Median length of stay, need for NiVS, ICU admission, MV, and IHM are shown in Table 2. The need for respiratory support diminished from the Delta to BA.4/5 period and average LoS shortened. In-hospital mortality dropped from approximately 17% in the Delta and BA.1 period to 10.3% in the BA.4/5 period.

**Table 2:** Length of hospital stay, in-hospital interventions and in-hospital mortality by predominant SARS-CoV-2 variant period.

	**Overall, *N =* 7,775^a^**	**Delta, *N =* 5,512^a^**	**Omicron BA.1, *N =* 1,120^a^**	**Omicron BA.4/5, *N =* 1,143^a^**	***p*-value^b^**
Length of hospital stay (days)	7 (4, 14)	8 (5, 15)	6.5 (3, 13)	5 (2, 9)	< 0.001
Non-invasive ventilatory support	1,393 (17.9%)	1,124 (20.4%)	180 (16.1%)	89 (7.8%)	< 0.001
Intensive care unit admission	1,043 (13.4%)	903 (16.4%)	111 (9.9%)	29 (2.5%)	< 0.001
Intensive care unit (hours)	299 (141.5, 538)	313 (157, 565)	218 (102, 426.5)	69 (12, 330)	< 0.001
Mechanical ventilation	828 (10.6%)	728 (13.2%)	80 (7.1%)	20 (1.7%)	< 0.001
Mechanical ventilation (hours)	328 (158.8, 574.2)	333 (168, 580.5)	205 (104.2, 443.8)	401 (125.5, 574.5)	0.019
**Mortality**					< 0.001
Other	6,543 (84.2%)	4,599 (83.4%)	919 (82.1%)	1,025 (89.7%)	
In-hospital mortality	1,232 (15.8%)	913 (16.6%)	201 (17.9%)	118 (10.3%)	

a n (%); Median (interquartile range).

b Pearson's Chi-squared test; Kruskal-Wallis rank sum test; Fisher's exact test.

**Figure 3:**
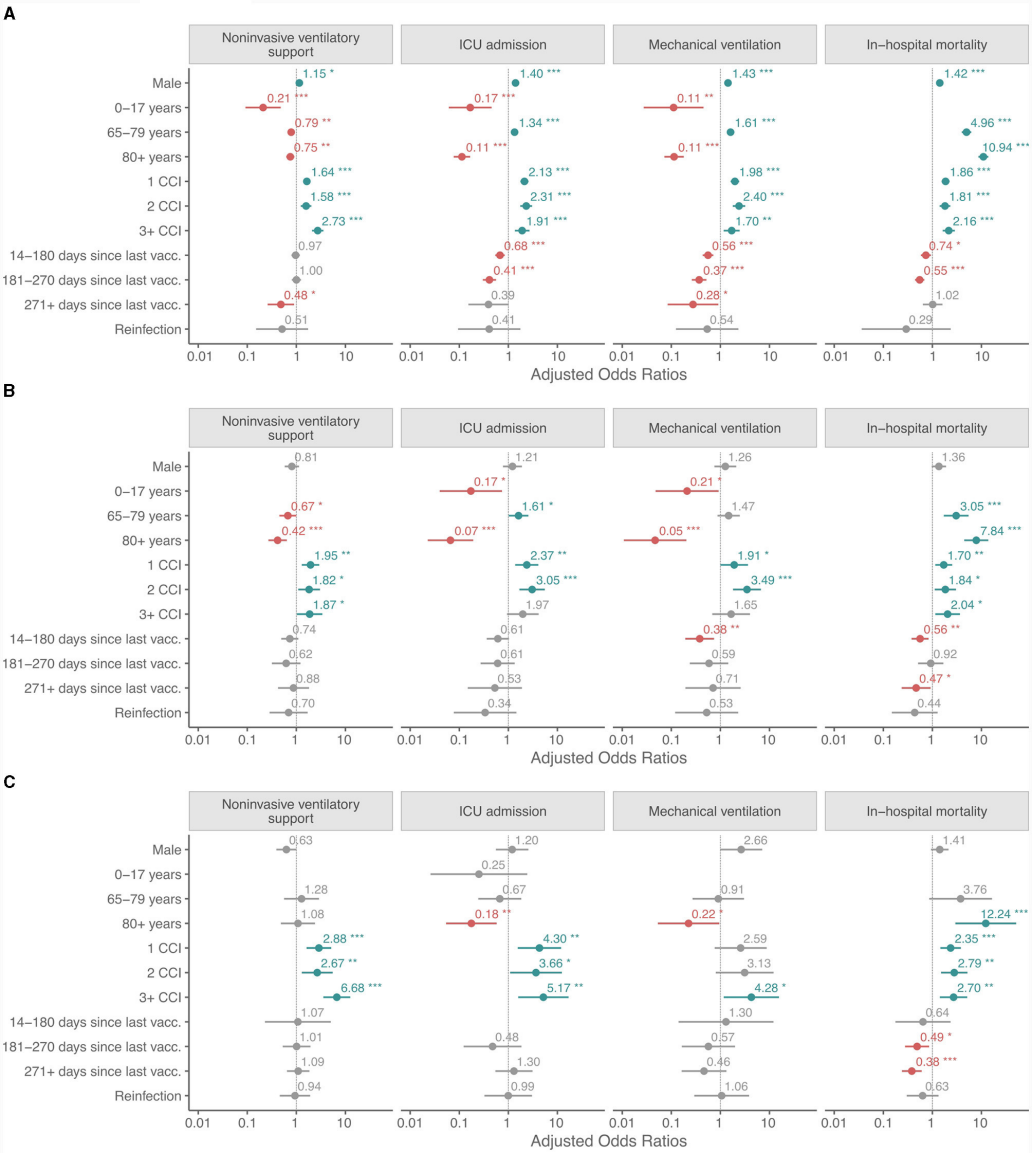
**(A–C)** Adjusted odds ratios for non-invasive ventilatory support, ICU admission, mechanical ventilation and in-hospital mortality in patients hospitalized for COVID-19 in the Delta, the Omicron BA.1 and BA.4/5 periods. Calculation is based on the following reference: sex: female; age group: 18–64 years old; CCI score: 0; time since last vaccine dose (for fully or additionally vaccinated individuals only): non-vaccinated or partially vaccinated; and reinfection: primary infection. *p < 0.05; **p < 0.01; ***p < 0.001.

Demographics, long term care residency, vaccination history, history of reinfections and comorbidities in patients who received NiVS, MV, were admitted to ICU or died during the hospitalization in the Delta, BA.1 and BA.4/5 periods were compared (Figures 3A–C and Supplementary Tables 2–5). The details of the univariate and multivariate logistic regression are available in Supplementary Tables 6–9. The variables included in the models were: sex (reference female), age (by groups) (reference 18–64 years old), CCI score (reference 0 CCI), time since last vaccine dose (for fully or additionally vaccinated individuals only) (reference non-vaccinated or partially vaccinated), and reinfection (reference primary infection). In multivariate analysis, men did not have statistically significant higher odds for NiVS, ICU admission, MV, or IHM, except in the Delta period (NiVS: OR: 1.15, 95% CI: 1.00–1.32, *p* < 0.05; ICU: OR: 1.40, 95% CI: 1.20–1.64, *p* ≤ 0.001; MV: OR: 1.43, 95% CI: 1.21–1.70, *p* ≤ 0.001; and IHM: OR: 1.42, 95% CI: 1.21–1.66, *p* ≤ 0.001) (Figures 3A–C). In comparison to 18–64-year old age group, patients aged ≥80 years had significantly lower odds for ICU admission and MV in all three periods. Odds ratios (OR) for ICU admission for ≥80 years old were in the Delta period: OR 0.11, CI 0.08–0.16 (*p* ≤ 0.001); B.A.1 period: OR 0.07, CI 0.02–0.17 (*p* ≤ 0.001); and BA.4/5 period: OR 0.18, CI 0.05–0.59 (*p* ≤ 0.004). The 65–79 age group had less consistent results—significantly higher odds for ICU admission [OR 1.34, CI 1.14-1.57 (*p* ≤ 0.001)], MV [OR 1.61, CI 1.35–1.92 (*p* ≤ 0.001)] and IHM in the Delta period and for ICU admission [OR 1.61, CI 1.03–2.58 (*p* ≤ 0.042)], and IHM, but not for MV [OR 1.47, CI 0.88–2.52 (p 0.147)] in BA.1 period. IHM was strongly related to increasing age in the Delta period (OR 10.9, CI 8.7–13.8, *p* ≤ 0.001), BA.1 period (OR 7.8, CI 4.6–14.3, *p* ≤ 0.001) and BA.4/5 period (OR 12.2, CI 3.7–75.6, *p* ≤ 0.001) in octogenarians and nonagenarians. To a lesser extent but still high odds with statistical significance for IHM were observed in 65–79 age groups in the Delta and BA.1 period (Figure 3 and Supplementary Tables 6–9). Multivariate analysis showed that time elapsed since last vaccination in fully/additionally vaccinated compared to non-vaccinated/partially vaccinated patients had no impact on NiVS except in the Delta period for those who were vaccinated 9 months before positive test (OR 0.48, CI 0.25- 0.87, p 0.022). Shorter time since last vaccine dose significantly decreased the probability for ICU admission in the Delta period (14–180 days since last vaccination: OR 0.68, CI 0.54–0.84, *p* < 0.001, 181–270 days since last vaccination: OR 0.41, CI 0.30- 0.56, *p* < 0.001, and ≥271 days since last vaccination: OR 0.39, CI 0.13- 0.92, p 0.053), but not in the Omicron BA.1 or BA.4/5 period. Statistically significant lower odds for MV and IHM were found in vaccinated compared to non-vaccinated/partly vaccinated in the Delta period regardless of time elapsed from last vaccine dose except for IHM in vaccinated more than 9 months ago. Time elapsed from last vaccine dose had an impact on MV and IHM in the Omicron BA.1 and BA.4/5 period as shown in Figure 3 and Supplementary Tables 6–9).

Prior SARS-CoV-2 infection had no statistically significant protective effect in regard to NiVS, ICU admission, MV and IHM in any of the variant periods.

A Charlson Comorbidity Index not equal to zero was associated with higher odds for NiVS, ICU admission, MV and IHM in the Delta, BA.1 and BA.4/5 periods, with few exceptions (ICU admission and MV for CCI ≥3 in the BA.1 period and MV for CCI 1 and CCI 2 in the BA.4/5 period).

**Figure 4:**
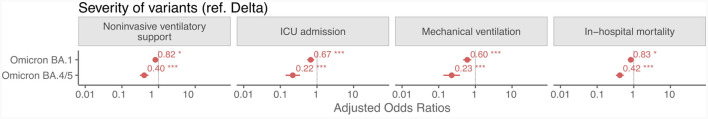
Adjusted odds ratios from logistic regression for non-invasive ventilatory support, ICU admission, mechanical ventilation and in-hospital mortality in patients hospitalized for COVID-19 in the Omicron BA.1 and BA.4/5 variants with respect to the Delta variant. *p < 0.05; **p < 0.01; ***p < 0.001.

Hospitalized patients with SARS-CoV-2 Omicron BA.1 and BA.4/5 were less likely to have NIVS, ICU admission, MV, and IHM compared to those with the Delta variant (Figure 4 and Supplementary Tables 10–13). Both Omicron variants had reduced progression to more severe disease, with BA.4/5 having lower odds than BA.1.

## 4 Discussion

We compared the characteristics of patients hospitalized in Slovenian acute care hospitals due to COVID-19 in the Delta, the Omicron BA.1 and BA.4/5 predominant periods. We found that the proportion of admissions due to COVID-19 decreased significantly during the period when the Omicron BA.1 variant was predominant compared to the Delta variant. The data from the present study are in accordance with the first studies from South Africa (1) and similar studies from different geographical areas and with different socio-economic and health systems (21, 22). The risk of hospitalization decreased from the Delta to the Omicron pandemic waves (3, 9–11, 13, 21, 23), even among older at-risk persons (12). Fewer studies have compared hospitalization rates, risk of hospitalization, differences in clinical severity and outcomes among patients infected with SARS-CoV-2 Omicron BA.1 and BA.4/5 variants (3, 4, 24–27). Studies from South Africa showed similar odds for hospitalization and severe outcome in BA.1 and BA.4/5 infected patients (3, 4). However, in both studies no distinctions were made between incidental, nosocomial and CAC COVID-19 hospitalizations. In contrast, a study from British Columbia, Canada found an 18% higher risk of hospitalization in BA.5 infected patients compared to BA.1 cases (24).

In the present study, the age structure changed noticeably from the Delta to the Omicron BA.4/5 waves. In the Delta wave, the majority of hospitalizations due to CAC were in the 18 to 64 age group, and only 2.1% were for children and adolescents. In the Omicron BA.4/5 wave, nearly half of the hospitalized patients were ≥80 years old, and one in eight admitted patients was under 18 years of age. The change in age structure with an increase in admissions of younger patients during the initial Omicron wave has been observed in studies of clinical severity and outcome in hospitalized patients infected during the Delta and the Omicron waves (1, 5, 28, 29). Jassat et al. (1) noted that the admission rate for people aged ≤20 years was higher in the Omicron wave than in the first three waves. The higher admission rate in children and adolescents could be attributed to the greater Omicron BA.1 transmissibility, lower rates of previous infection, lower vaccination rates, and a greater number of incidental infections in children hospitalized for other reasons (1). In the present study, patients with nosocomial COVID-19 or admitted for other reasons and incidentally positive for SARS-CoV-2 were excluded from the analysis. It might be that Omicron causes more symptomatic acute respiratory infection demanding in-hospital treatment in children. Sumner et al. noticed that children with Omicron variant infection who presented to the emergency department were more likely to have fever and lower respiratory tract symptoms compared to previous variants (30). Interestingly, other studies found no difference in the risk of hospitalization among school-aged children (5–12 years) infected with Omicron relative to Delta, and no increase in hospitalization in children younger than five (13, 31).

The current study shows that the sex structure of the CAC changed between the study periods. Significantly more female patients were admitted in the BA.4/5 wave compared to the Delta and BA.1 wave. Earlier studies comparing the sex distribution of hospitalized patients showed conflicting results, with an increase in the female-to-male ratio from the Delta to the Omicron BA.1 or BA.4/5 periods in some publications (3, 25, 32), a decrease in others (7) or no change at all (33). Various societal factors should be taken into consideration to explain the demographic differences. During the Omicron wave most of the non-pharmaceutical measures were relaxed or only recommended, but not followed with the same rigor as in earlier waves. As such, the number of outbreaks in nursing homes increased and a higher proportion of hospitalized patients were residents of long-term care facilities in the Omicron period. In Slovenia, 0.9% of the population resides in long-term care facilities with a female-to-male ratio of 3:1 (https://www.stat.si/statweb/News/Index/8374), which may partially explain the increase in the number of women hospitalized during the Omicron waves. It may also be that hospital admission policies were changed and a lower clinical threshold for the admission of young children with respiratory symptoms and nursing home residents was introduced.

In Slovenia, the vaccination COVID-19 policy (as in other countries) was that the most vulnerable (nursing home residents, older adults and persons with comorbidities) received COVID-19 vaccine first along with health-care workers. The vaccination started in the end of 2020 with highest intensity in spring 2021 as shown in Figure 2. The vaccination coverage rate increased from the Delta to the Omicron waves. In the BA.4/5 period, 44.1% of hospitalized patients had already received a booster mRNA vaccine dose. The data reflect vaccination dynamics in Slovenia—from the end of October 2021 to mid-January 2022 about a quarter of the Slovenian population received an additional dose (webpage: https://vaccinetracker.ecdc.europa.eu/public/extensions/COVID-19/vaccine-tracker.html#uptake-tab). After spring 2022, interest in COVID-19 vaccination dropped drastically, with relatively a small increase in the number of newly vaccinated people. As a result, the average time elapsed since the last vaccine dose was shortest for hospitalizations in the Omicron BA.1 wave and longest in the Omicron BA.4/5 wave. Only 3.4% of those hospitalized with BA.4/5 CAC had been vaccinated in the last 6 months before testing positive for SARS-CoV-2 and being hospitalized for CAC. The increase in the number of vaccinated among hospitalized CAC patients with BA.4/5 infection might be associated with a decrease in the degree of protection against the BA.4/5 variant and waning vaccine derived-immunity. Studies have shown that vaccination was associated with a reduction in disease severity for infections with either the Delta or Omicron BA.1 or BA.2 variants (3, 11, 13, 22), with the difference being more pronounced for infections with the Delta variant (14). In the present study, multivariate analysis revealed significantly lower odds of ICU admission and IHM in the Delta period for those who were fully vaccinated or additionally vaccinated within last 9 months. Vaccination lowered the odds of MV regardless of the time elapsed between the last vaccine dose and positive test result for COVID-19. In the BA.1 variant period, patients with less than 6 months elapsed between the last vaccine dose and SARS-CoV-2 positivity had lower odds of MV and IHM, but not of NiVS or ICU admission. Vaccination with a COVID-19 vaccine had no effects on NViS, ICU admission, or MV in the BA.4/5 period, but decreased the probability of IHM.

In the present study a substantial surge in the rate of reinfections was observed in the Omicron BA.1 and BA.4/5 waves compared to the Delta wave. The proportion of asymptomatic infections is unknown but most probably under-ascertained (18, 19), and we believe that the same holds for our study, with the reinfection rates underestimating the real number of reinfected and hospitalized people. After 2 years of the epidemic the level of testing for SARS-CoV-2 infection was dramatically reduced in Slovenia, rendering the estimation of the true proportion of those who had been infected at least once before a SARS-CoV-2 infection that required hospitalization difficult. A population-based study carried out in the country showed that prior to the Omicron BA.4/5 predominant wave, there was a 35.5% seroprevalence of SARS-CoV-2 anti-N antibodies in participants who had never been officially notified as infected (34).

The categorized and weighted comorbidities according to Charlson (and Quan modification) showed a substantial increase in CCI from the Delta to Omicron BA.4/5 waves, similar to the results of a study carried out in the United States, in which adult hospitalized patients tended to have more risk factors for severe COVID-19, i.e., they were on average older, had underlying chronic medical conditions and/or were immunocompromised during the Omicron BA.1, BA.2, and BA.4/5 waves compared to previous waves (25). Nevertheless, disease severity decreased as measured by systemic inflammation, coagulopathy, early discharge, reduced need for ventilatory support and death. The lower severity of the BA.4/5 variant compared to the BA.1 and the Delta variants was confirmed in the present study. Studies published on the severity of BA.4/5 found no difference, increase or decrease compared to the earlier Omicron variants (3, 24, 26, 33, 35, 36). However, these conflicting results merit further research as do studies regarding quality of life indicators and their correlation with the severity of the SARS-CoV-2 infection (37).

The present study has several strengths. For one, Slovenia has good and reliable national registries, and by linking national databases on hospitalizations and vaccinations for people who tested positive for SARS-CoV-2 we ensured the completeness of the data. Second, compared to other countries a very large proportion of sequenced isolates were reported to GISAID throughout the course of the pandemic, and therefore with variants' periods of predominance could be reliably defined. Third, we compared the COVID-19 severity in patients hospitalized for community-associated Delta, BA.1 and BA.4/5 variant infection. In many (but not all) previous studies the distinction between hospitalized with or because of COVID-19 has not been made. Patients with other health problems requiring hospitalization and patients incidentally infected with SARS-CoV-2 were excluded from our study as were patient with HAI. Hospital-acquired COVID-19 represented a serious public health issue and have been already addressed in previous studies (38, 39). The present study has several limitations. First, the main discharge and additional diagnoses were obtained from health-statistic data sources. It is possible that a complete list of chronic diseases and/or conditions was not recorded at discharge, resulting in an underestimated comorbidity. However, we assume that the incompleteness of records was approximately the same across epidemic waves, and cannot be the cause of the detected differences in the Charlson index. Second, patients were classified as infected with the Delta, BA.1, or BA.4/5 variants according to most likely variant inferred from the dominant strain in Slovenia at that time. Therefore, some degree of uncertainty remained in the classification used. Third, the present study is a retrospective observational study with possible residual confounding. While multivariate models were used to address this, not all differences could be corrected for. The vaccination might have an impact on milder course of COVID-19 in the Omicron wave compared to the Delta wave but even in populations with low vaccination coverage e.g. in the Republic of South Africa, where <25% of the adult population was vaccinated, the same phenomenon has been observed (40). There are other limitations of the study that deserve to be mentioned. Viral load is an important factor which might influence the course of the disease. In our study, we did not have data on viral load in patients hospitalized for COVID-19, thus we could not study the correlation with disease severity. There is a possibility that co-infections of SARS-CoV-2 with influenza or RSV viruses were missed, because they were not mentioned among the discharge diagnoses. We assume that such cases were rare due to the low intensity of the influenza and RSV season in 2021 and the first half of 2022. We do not have an information on various treatments prescribed to patients who were hospitalized because of COVID-19. Therefore, we could not include the various treatments in the analyses, which is one of the limitations of the study.

## 5 Conclusions

The findings of this study suggest decreased severity of the Omicron BA.4/5 variant compared to the BA.1 and Delta variants. Vaccination had the greatest effect in preventing severe COVID-19 in the Delta wave, and in the Omicron BA.4/5 wave it reduced the likelihood of intra-hospital death. Surveillance using affordable testing strategies (e.g., sentinel and Severe Acute Respiratory Infections (SARI) surveillance) should continue to provide timely information on the development of new SARS-CoV-2 variants and changes in clinical severity.

## Data availability statement

The data analyzed in this study is subject to the following licenses/restrictions: The datasets are available in accordance to the national legislation. Requests to access these datasets should be directed to MM, maja.mrzel@nijz.si.

## Ethics statement

The studies involving humans were approved by Committee for Deontological and Ethical Issues, NIPH. The studies were conducted in accordance with the local legislation and institutional requirements. Written informed consent for participation was not required from the participants or the participants' legal guardians/next of kin in accordance with the national legislation and institutional requirements.

## Author contributions

MS: Writing – original draft, Writing – review & editing. MM: Writing – original draft, Writing – review & editing. KP: Writing – review & editing. MK: Writing – review & editing. TA-Ž: Writing – review & editing. MP: Writing – review & editing. ML: Writing – review & editing. TZ: Writing – review & editing.
